# Synthetic short RNA in cancer

**DOI:** 10.3389/fonc.2026.1821729

**Published:** 2026-05-28

**Authors:** Takeshi Tomita, Kentaro Minagawa, Sachie Hiratsuka

**Affiliations:** 1 Institute for Biomedical Sciences, Research Cluster for Social Implementation, Shinshu University, Matsumoto, Japan; 2 Department of Biochemistry and Molecular Biology, School of Medicine, Shinshu University, Matsumoto, Japan; 3 Research Center for Cell and Gene Therapy, Shinshu University, Matsumoto, Japan; 4 Hematology & Oncology, Penn State Cancer Institute, Hershey, PA, United States

**Keywords:** antisense oligodeonucleotides, aptamer, extracellular RNA, hybridization-dependent function, hybridization-independent function, siRNA

## Abstract

Small RNAs play an important role in many biological processes. They vary widely in size and form, including single- and double-stranded RNAs. Synthetic short RNAs are very powerful tools for modulating or intervening in cellular biology, and many types of artificial oligonucleotides have been explored to mimic small RNAs. For instance, small interfering RNA (siRNA) hybridizes with target RNA to decrease protein expression by promoting RNA degradation. An aptamer binds to target molecules through non-hybridizing nucleotide-molecule interactions. Furthermore, recent findings suggest that synthetic nucleic acids utilizing extracellular mRNA sequences act on immune cells expressing RNA-binding molecules, resulting in anti-tumor effects. These molecular specificities are generated by the primary sequence of the nucleic acid and its chemical modifications. These features are applied to anti-tumor drug discovery. In this review, we summarize the basics of synthetic short RNAs and discuss their anti-tumor potential.

## 1 Introduction

In recent decades, the population of cancer patients has grown, and this trend is expected to continue. Thus, the social concern is to stop this trend. Although the molecular mechanisms governing cancer biology are not yet fully understood, advances in biochemical science have found that usefulness of extracellular RNAs to manipulate tumor cells or the tumor microenvironment. Thus, many synthetic short RNAs have been tested as cancer treatments. Compared with other modalities, such as chemical compounds, peptide derivatives, and antibodies, RNA-based therapy is characterized by high versatility and druggability (1). RNA-based compounds are used for protein production (mRNA vaccines), gene silencing (siRNAs), and triggering the cleavage of target RNA or modulating its splicing (antisense oligonucleotides). On the other hand, RNA-based therapy has disadvantages regarding stability, delivery, and renal clearance. Currently, an oligo synthesizer can produce synthetic short RNAs at lengths of approximately 100 mer. Introducing nucleotide sequence variations and/or chemical modifications enhances stability and efficacy. The chemical structure of modified RNA resembles that of DNA because the 2’-OH group in the sugar moiety of RNA is often replaced with another group to increase stability. In this review, we define synthetic short RNA as DNA- or RNA-based synthetic oligonucleotides less than 100 mer in length. In terms of the advantages of RNA-based therapy, *in vitro* sequence selection techniques, such as SELEX (Systematic Evolution of Ligands by Exponential Enrichment) (2–4), are extraordinarily powerful because they allow us to simultaneously screen a large number of primary oligonucleotide sequences to identify the optimized sequence. This advantage is particularly remarkable when a new molecule is designed to recognize a target molecule, such as an antibody. Thus, producing a new aptamer is much easier and less costly than producing a monoclonal antibody. In this review, we first present an overview of small RNAs in biology because the concept of synthetic short RNA was derived from them. We then summarize the details of each synthetic short RNA and discuss recent progress in the mRNA type of synthetic short RNA (synthetic short mRNA). This is a new category, being expected to grow because synthetic short mRNA is a powerful tool for combating cancer.

## 2 Small RNA in biology

Several types of small RNAs play important roles in many biological processes. They are transcribed from the genome and processed by a unique system. Endogenous small interfering RNA (siRNA) is rare in mammals (5–7) because siRNA biogenesis begins with double-stranded RNA, but the key enzyme for producing siRNA, RNA-dependent RNA polymerase (RdRp), is not present in mammalian cells. Nevertheless, mammalian cells have proteins that process exogenous siRNA, including Drosha, which allows it to function in the cells (8). Finally, siRNA binds to its target transcript in a sequence-dependent manner, facilitating its degradation. In contrast, microRNAs (miRNAs) are encoded in the genomes of mammals. The nascent transcript of a miRNA is designated as pri-miRNA. It is processed by the Drosha/Pasha complex to generate a pre-miRNA (9). The complex is then transported to the cytoplasm, where it is further processed by the Dicer complex to produce mature miRNAs. The RNA-induced silencing complex (RISC) binds the mature miRNA, and Ago2 dissociates the duplex structure, making it bioactive. Finally, the single-stranded miRNA binds to a target transcript in a sequence-dependent manner to suppress its translation. The PIWI-interacting RNA (piRNA) (10) precursor is transcribed from single- or double- stranded piRNA clusters and is processed into piRNA intermediates that bind PIWI proteins to form the piRNA complex. This complex silences transposons in the nucleus. It has also been reported that piRNA complexes exert antiviral and epigenetic effects. Small nucleolar RNA (snoRNA) (11, 12) is present in the nucleolus and facilitates the modification of rRNA. These modifications, including 2’-O methylation and pseudouridylation, are essential for the maturation of rRNA. Small nuclear RNA (snRNA) (13) is a component of the spliceosome. Precursor snRNAs are first transcribed and processed to create mature forms. Mature snRNAs function to splice introns out of pre-mRNAs in the spliceosome, creating mature mRNA. Transfer RNA-derived fragments (tsRNAs) (14, 15) are generated by specific cleavage of tRNAs. Initially, tsRNAs were regarded as waste, but it was later found that they are involved in a wide variety of biological processes, including the regulation of mRNA stability and regulation of protein translation. Y-RNA (16, 17) is a small non-coding RNA that provides a scaffold for pivotal biological processes, including DNA replication and RNA quality control.

## 3 Synthetic short RNA

**Figure 1:**
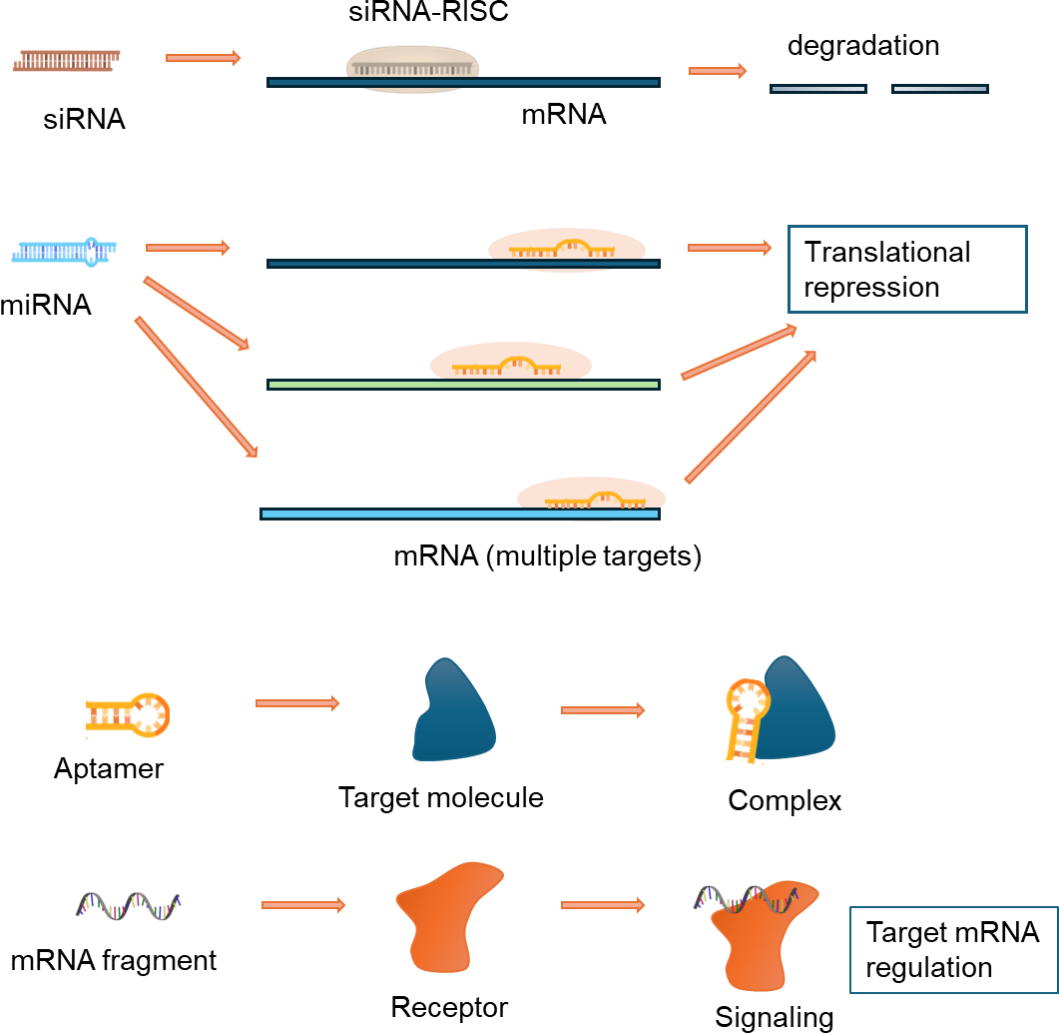
Hybridization-dependent and -independent interactions of synthetic short RNAs. Hybridization-dependent interactions: siRNA binds target mRNA through base-pair interactions. Similarly, miRNA binds multiple mRNAs because its binding specificity is not as strict as siRNA. Hybridization-independent interactions: Aptamers with optimized sequences have high affinity for target molecules. mRNA fragment interacts with its receptor protein to regulate gene expression in a sequence-dependent and hybridization-independent manner. Part of the image is taken from TogoTV (^©^ 2016 DBCLS TogoTV, CC-BY-4.0 https://creativecommons.org/licenses/by/4.0/deed.ja.

Many synthetic short RNAs are designed to perform the biological activities of small RNAs, and their nucleotide sequences are derived from naturally occurring small RNAs. Additionally, synthetic short mRNA is emerging as a new category (**Figure 1**). Because mRNA is not considered to function as a signaling molecule in the extracellular sphere, it has not been widely explored (18). Recently reported synthetic short mRNA is designed to contain a nucleotide sequence within the functional region of non-vesicular extracellular mRNA (19). Furthermore, some synthetic short RNAs with fully artificial sequences, including aptamers, are used to confer specific functions on oligonucleotides, such as protein or small-molecule recognition and the degradation of target RNA. Nowadays, the preparation of synthetic short RNA has become relatively easy. Given the many variations in length and chemical modification, we can design new oligonucleotide types to study their distinct biological functions.

### 3.1 Chemical modifications

**Figure 2:**
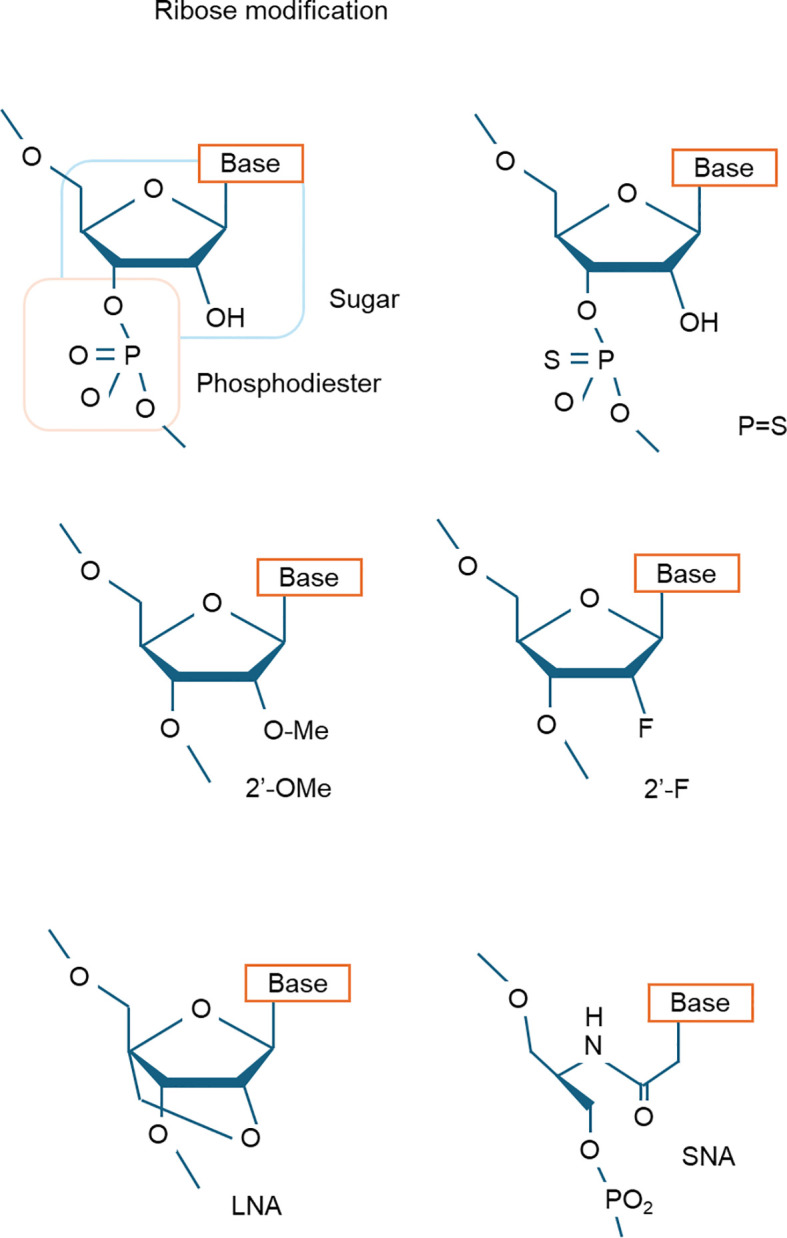
Ribose modifications for synthetic short RNAs. Chemical structure of RNA, phosphorothioate, 2’-Ome, 2’-F, and LNA are shown. In addition, the structure of SNA, one of the well-characterized acyclic nucleotide analogues, is also shown.

The character of a synthetic oligonucleotide is determined by its sequence and modifications. Both naturally occurring and artificially designed modified nucleotides can also be incorporated into oligonucleotides. Many modifications have been applied to various biological systems to date. These modifications are classified into three groups: base, sugar (ribose), and phosphodiester modifications. Nucleic acid analogues such as morpholino (20, 21) and acyclic nucleic acids such as serinol nucleic acid (SNA) and L-threoninol nucleic acid (L-aTNA) (22) can also be incorporated (**Figure 2**). These modifications can be applied individually or in combination to achieve optimal results, as outcomes may vary on a case-by-case basis. First, these modifications affect the thermostability of oligonucleotides (23). RNA is an unstable molecule because it is single-stranded, and the 2’-OH group of the ribose moiety is highly reactive. Thus, modifications to the 2’-position of ribose improve the stability of RNA. Second, it is well known that the chemical modifications increase the RNase resistance of oligonucleotides. Synthesized single-stranded RNA with no modifications degrades rapidly in the presence of serum because serum contains RNases. Introducing a modification at the ribose 2’- position effectively prevents the degradation, but solely 2’-O methylation alone is insufficient for complete stability (24). Additional modifications should be introduced at the 5’- and 3’- ends of the nucleotides to increase stability when exposed to RNase over an extended incubation period (25). Third, chemical modifications can also influence the biological properties of an oligonucleotide. Single-stranded 2’-O-methyl modified RNA is recognized by the TLR7 receptor but does not induce the expression of inflammatory cytokines (26). It was shown that 5’-methyl-C modifications provide an additional function in self-amplifying RNA transfection (27). Self-amplifying RNA containing 5’-methyl-C modifications in all cytidines reduces the inflammatory responses when transfected into various cells. This RNA encodes RdRp, and the nucleotide modifications of the original template RNA are transferred to the nascent RNAs when amplified by RdRp. Consequently, all amplified RNA contains m5C modifications. Transfection with m5C-modified self-amplifying RNA resulted in high-level expression of proteins and low-level expression of IFNs (27). Thus, these facts indicate that modified oligonucleotides are expected to have a longer biological half-life and fewer adverse effects than their unmodified counterparts. It was reported that introducing locked nucleotide (LNA) into an antisense oligonucleotide (ASO) renders it RNase-resistant, but this modification causes significant hepatotoxicity (28). Later, the same research group found that hepatotoxicity occurs in a sequence-dependent manner (29). It is well known that phosphorothioate modification (P=S) increases cellular uptake (30). Therefore, most ASOs used for therapeutic purposes are administered in a naked state without being incorporated into lipid nanoparticles. Lipid modification at either end of an ASO also improves cellular uptake. Specific ligand attachment, such as GalNac modification at the 5’-end, increases liver-specific delivery (31). However, increased delivery does not necessarily indicate greater efficiency of the incorporated ASO, as modification may adversely affect its intracellular function. Biscans and collaborators demonstrated that phosphorothioate modification of the phosphodiester group of siRNA increased cellular uptake, though high P=S content reduced gene knockdown efficiency (32). The addition of a lipid increased cellular uptake but reduced its interference ability. This reduction could be avoided by inserting a cleavable linker at the 3’ end of the guide strand, which implies that lipid modification inhibits RISC complex formation. This is because siRNA requires a phosphate group at the 5’ end to be recognized by RISC (32).

### 3.2 Sequence specificity

There are two types of sequence-specific molecular interactions: hybridization-dependent and hybridization-independent. Hybridization-dependent interactions of siRNA: For an oligonucleotide, the interaction with the largest free energy is the formation of a duplex, where the base pairing is the main driving force. In this case, sequence matching is essential, and mismatches are usually not tolerated. Therefore, siRNA usually requires perfect matching to the target sequence. However, both ends (1^st^ position base, that is 5’ end, and 18^th^ and 19^th^ positions base, that is, 3’ end) are relatively tolerant of mismatches (33). Liang, Du, and collaborators demonstrated that siRNA sequence matching is more tolerant of mismatches when the target sequence is in the 3’UTR region (34). It is well known that siRNA can have an off-target effect, which is derived from the matching of the seed region (2^nd^-8^th^ position nucleotides) of the guide (antisense) strand or passenger (sense) strand to non-target mRNAs (35, 36). Hybridization-independent interactions of siRNA: It was reported that introducing a chemical modification that creates steric hindrance can drastically reduce the seed-matched off-target effects (37). Furthermore, the cellular uptake and overall knockdown efficiency of various combinations of guide and passenger strands were tested (32). The addition of 5’-overhang (2 nt) in the passenger strand increased cellular uptake compared to the blunt format, but a longer overhang (5 nt) reduced the effect. On the other hand, the gene knockdown ratio of the 5-nt overhang was higher than that of the 2-nt overhang (32).

Hybridization-dependent interactions of miRNA: miRNA functions by binding to target mRNA in a primary sequence-dependent manner. Unlike siRNA, mammalian miRNAs do not necessarily have a perfectly matching sequence on the target mRNA; they bind to a partially matching sequence, which leads to the suppression of translation and the deadenylation of mRNA. This partial matching requires base-pair interactions in the miRNA seed region (2^nd^-8^th^ positions) (38) and supplementary region (13^th^-16^th^ positions) (39), so these regions must nearly perfectly match the target mRNA, while 10^th^-12^th^ positions should have a mismatch or form a bulge structure. These facts indicate that the sequence specificity of miRNA against target mRNAs is not as strict as that of siRNAs. In addition, a single mRNA can bind multiple miRNAs simultaneously, and a single miRNA can interact with multiple mRNAs. Thus, there are many possible combinations between miRNAs and mRNAs. To address this complexity, deep learning-based prediction methods for miRNA-mRNA interactions have been proposed (40, 41).

Hybridization-independent interactions of aptamer: An aptamer interacts with its target molecule independently of hybridization. In the case of a protein-recognizing aptamer, protein-nucleotide interactions drive the aptamer’s affinity. The stringency of the primary nucleotide sequence depends heavily on protein-nucleotide interactions, and an optimized aptamer can outperform an antibody (42). Hybridization-independent interactions of short mRNA: Regarding synthetic short mRNA, the receptor protein recognizes it, so protein-nucleotide interactions are pivotal in determining its molecular specificity. An RNA-binding protein can bind to a consensus sequence, such as an AU-rich element. In an *in vitro* SELEX study, Jolma and collaborators showed that optimizing the binding RNA for AU-rich element-binding proteins revealed a wide variety of RNA sequences (43). These results imply that the stringency of RNA sequence specificity is not fully understood. However, several deep learning-based RNA-protein binding predictions have been proposed (44, 45). In the case of synthetic short mRNA, the RNA fragment captured by the receptor may interact with different proteins inside cells. Thus, the fragment is expected to interact with multiple proteins through regions other than the AU-rich element.

### 3.3 Structure

RNA molecules carry more biological information than their primary sequence alone. In the biological machinery, RNA molecules interact with RNA-binding proteins, and these interactions are modulated by the structural factors of RNA. Notably, not only rigid structural domains in proteins, but also disordered regions, can interact with RNAs (46). Therefore, it is difficult to predict protein-RNA binding style based on protein structure alone; information on RNA structure is also necessary. The first step in analyzing RNA structure is evaluating its secondary structure. Three well-known secondary structures -the helix, stem loop, and pseudoknot- are generated by internal base-pairing interactions of single-stranded RNA. The experimental determination of RNA structures is laborious and costly. However, a computational approach has been developed to address this issue. Various computational methods have been proposed, and classical methods are based on thermodynamic calculations to minimize free energy, with base pairing as the most important factor. Because classical methods are not well suited to calculating complex secondary structures such as pseudoknots, recently proposed methods employ deep learning to improve the accuracy of RNA secondary structure prediction. UFold (47) and RNADiffFold (48) demonstrated high accuracy in analyzing test RNA datasets. KnotFold (49) also demonstrated high performance in predicting pseudoknot-containing structures. The same trend applies to RNA 3D structure prediction. RhoFold+ (50), trRoseTTARNA (51), and NuFold (52) are among the most accurate methods. In addition, AlphaFold3 (53) was recently released. It is an extension of the renowned protein structure prediction method AlphaFold2 (54) and can predict the structures of molecules other than proteins. While these structure prediction methods are excellent, they are not suitable for analyzing chemically modified structures and/or molecular-molecular interactions because they do not consider these additional factors (55). Therefore, experimental structure determination remains advantageous for analyzing protein-RNA interactions over computational prediction methods. In a cryo-electron microscopy study, Toor and co-workers proposed a new method to analyze the structure of short RNAs to overcome the difficulty of determining RNA’s 3D structure. In this study, short RNA (thiamine pyrophosphate riboswitch) was combined with a large RNA with a known structure (Group II intron) and analyzed as a single molecule to determine structural changes that occur upon ligand binding. This was accomplished by comparing ligand-bound and ligand-free structures (56).

## 4 Synthetic short RNAs in clinical trial

**Table 1** summarizes the synthetic short RNAs that have been tested in clinical trials. The table does not include several mRNA-type RNA-based drugs because they are long enough to encode the target protein(s). The limited number of synthetic short RNAs listed in the clinical trials indicates translational barriers. This review discusses these barriers from a biological viewpoint.

**Table 1:** Synthetic short RNAs tested in clinical trials.

Candidate Name	RNA Modality	Target / Mechanism	NCT Number(s)	Lead Indication	Current Phase
RAG-01	saRNA	Activates p21	NCT06351904	NMI Bladder Cancer	Phase 2
siG12D-LODER	siRNA	KRAS G12D/G12V mutations	NCT01676259	Locally Advanced Pancreatic	Phase 2
OT-101	ASO	TGF-β2	NCT06079346	Pancreatic Cancer, Glioblastoma	Phase 2b/3
BP1001	ASO	Grb2	NCT02923986	AML,CML, Solid Tumors	Phase 2
BP1002	ASO	Bcl-2	NCT04072458	AML, Lymphoma	Phase 1/2
Danvatirsen	ASO	STAT3	NCT02983578	DLBCL, NSCLC, Head & Neck Cancer	Phase 2
IONIS-AR-2.5Rx	ASO	Androgen receptor	NCT02144051	Metastatic Prostate Cancer	Phase 1/2
AZD4785	ASO	KRAS	NCT03101839	Advanced Solid Tumors, NSCLC	Phase 1
NOX-A12 (Olaptesed pegol)	aptamer	CXCL12 (SDF-1)	NCT04121455 NCT03168139	Glioblastoma, Pancreatic Cancer	Phase 1/2
AST-201	aptamer	GPC3 (Glypican-3)	NCT06687941	Liver Cancer	Phase 1
AS1411	aptamer	Nucleolin	NCT00740441 NCT01034410	AML, Renal Cell Carcinoma	Phase 2

The first hurdle for RNA-based drugs is drug delivery, especially if they are modified to be resistant to RNase degradation. Free short RNAs are rapidly filtered by the kidneys, while short RNAs encapsulated in nanoparticles are trapped in the liver. This indicates that a higher dose is required to deliver RNA-drugs, particularly to extrahepatic tissues. This leads to liver toxicity (57). Another hurdle for RNAs carried in lipid nanoparticles is endosomal escape. Typically, only 1-2% of RNA typically escape the endosome when the nanoparticles are taken into the cells (58). Finally, immunological side effects, such as cytokine storm, may occur because the immune system has machinery that responds to exogenous RNAs (59). In addition, there may be an adverse effect from excessive knockdown of the hybridizing gene or from affecting the other genes via partial complementarity.

## 5 Antitumor synthetic short RNAs

### 5.1 siRNA, miRNA, and various ASOs

The advantages of siRNA- and ASO-based drugs are their high specificity for the target gene and their ability to efficiently silence it. A miRNA-based drug is beneficial if it can target multiple genes simultaneously. While there are some FDA-approved RNA-based drugs, none are for cancer treatment. However, many siRNA- (60), miRNA- (61, 62), and ASO- (63, 64)based cancer drugs are currently being tested. These drugs target a wide variety of proteins, including transcription factors and intracellular signaling molecules that play important roles in maintaining tissue homeostasis. In addition, anti-miRs, which are complementary sequences that block the biological functions of miRNAs, are a potential new class of drugs for cancer treatment. For example, antimiR155 conjugated with a peptide that forms a transmembrane structure in response to low pH inhibited the tumor growth of mouse lymphoma cells (65). Small activating RNA (saRNA) is a duplex RNA that activates target gene expression (66). SiRNA and saRNA have the same chemical structure, and some miRNAs serve as saRNA. Thus, AGO2 binds to saRNA, and the resulting complex is transferred into the nucleus. The AGO2-saRNA complex binds to the promoter region of the target gene in a hybridization-dependent manner, leading to the activation of transcription. MTL-CEBPA, a saRNA targeting CEBPalpha, is expected to be an effective drug for treating liver cancer (67). Splice-switching RNA is also a promising molecule in this category (68, 69). It binds to nascent mRNA transcripts to control their splicing patterns. Depending on the annealing position of this short RNA, a specific exon is excluded or included in the final mRNA product. This technique can generate a readthrough (70), or include or exclude a poison exon (71) or a premature termination codon-containing exon. mRNA containing a premature termination codon is rapidly degraded by nonsense-mediated decay. It is reported that a splice-switching oligonucleotide that alters the splicing of the RE1-silencing transcription factor (REST) to deprive the REST protein of its biological functions inhibits the proliferation of small cell lung and neuroendocrine prostate cancer cell lines (72). The researchers also demonstrated the anti-cancer effects of the splice-switching oligonucleotide by using a xenograft mouse model (72).

### 5.2 Aptamer

Aptamer-based drugs have several advantages. They are smaller than antibodies, and the synthesis and modification of aptamers is much easier than that of protein-based drugs. Aptamers are expected to exhibit high specificity and affinity for their target molecules. In addition to empirical screening, such as SELEX, it is possible to use computational screening to optimize the aptamer sequence to recognize the target molecule (73). Currently, only small number of aptamers are being tested in clinical trials for cancer treatment (73, 74). AS1411 is among the most well-studied aptamers in clinical trials. It has a 26-mer sequence, 5’-GGTGGTGGTGGTTGTGGTGGTGGTGG-3’, which forms a G-quadruplex structure (75) and inhibits glioma proliferation (76). This aptamer is recognized by the cell surface nucleolin protein, and upon nucleolin binding, AS1411 is taken up by the recipient cell (75). In a structural study, Bie and collaborators proposed that the RBD1 and RBD2 domains of nucleolin pinch the G-quadruplex (77). Olapseted pegol contains a 45-mer L-stereoisomer oligonucleotide that binds to CXCL12 (78), which plays an important role in cancer progression (79). Thus, Olapseted pegol has been tested in clinical trials for metastatic colorectal and pancreatic cancers and multiple myeloma (73). Since aptamers can be modified to recognize cell-surface receptors like antibodies, the aptamer conjugation technique can deliver anti-tumor drugs, lipid nanoparticles, or siRNAs to cancer cells. Sgc8c aptamer, when conjugated with the chemotherapeutic drug (80) is useful for treating cancer with PTK7, a receptor tyrosine kinase. Since many malignant tumor cells overexpress PTK7 and sgc8c binds PTK7, the sgc8c conjugate can effectively deliver the tumoricidal drugs. MA3 aptamer conjugated with doxorubicin can kill human lung and breast cancer cells because the 86-mer specifically binds MUC1 protein (81). EpCAM aptamer, when combined with mesoporous silica nanoparticles loaded with doxorubicin, was shown to kill colorectal cancer cells (82). In addition, its antibody-like properties are being used to develop aptamer-based cancer immunotherapies (83). CTLA-4 aptamer specifically blocks the cell surface CTLA-4 receptor and promotes the antitumor activity of CD8^+^T cells. It was also demonstrated to suppress tumor growth in the mouse model study (84). Pegylated MP7 aptamer specifically binds to PD-1, blocking the PD-1/PD-L1 interaction and suppressing the growth of MC38 colon cancer cells expressing the human carcinoembryonic antigen in C57BL/6 mice (85).

### 5.3 Synthetic short mRNA

**Figure 3:**
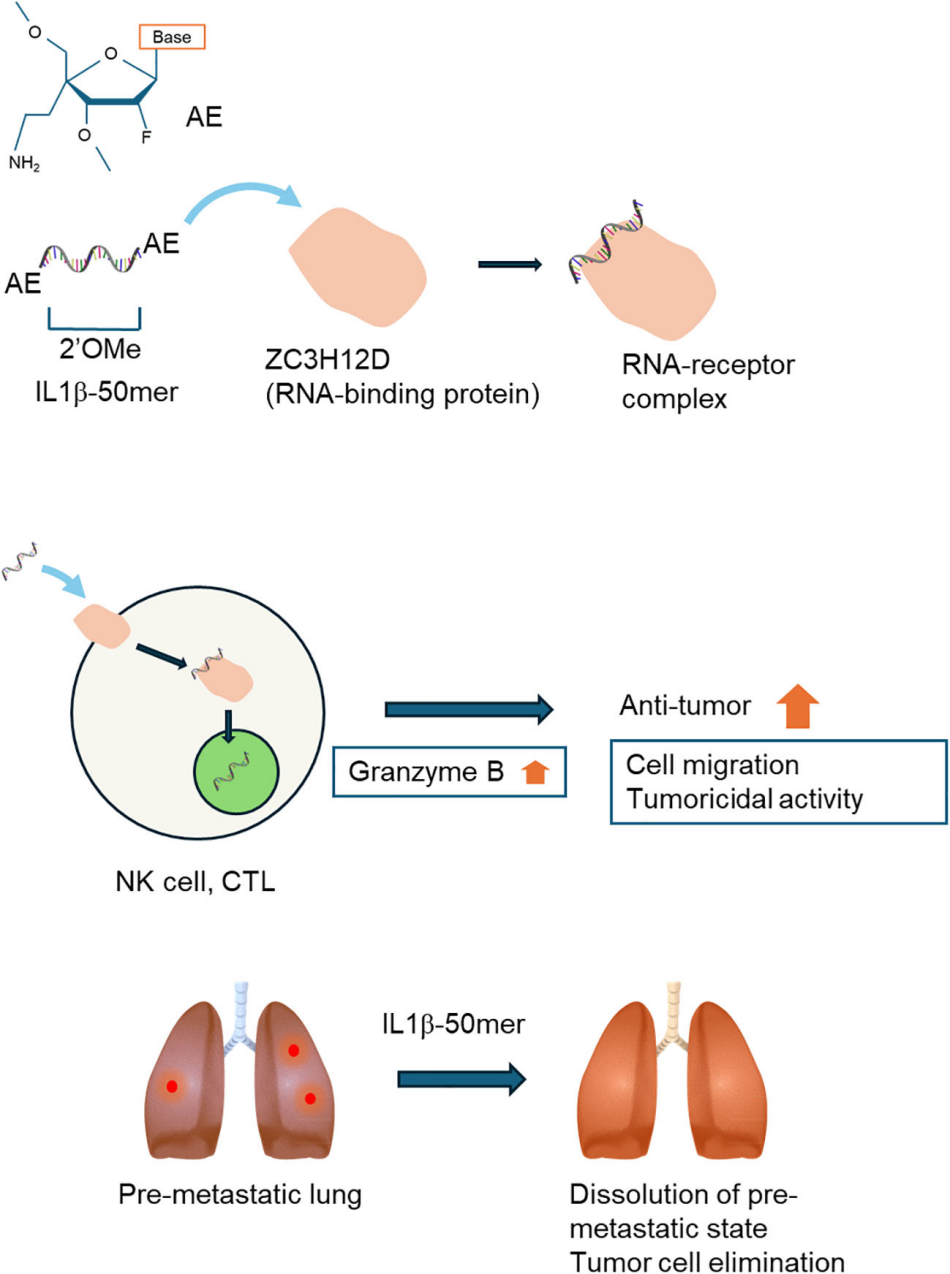
Therapeutic usage of synthetic short mRNA. Synthetic mRNA, with AE modification at both ends and 2’-OMe modifications for the 48 nucleic acids in between, was used for the ligand of ZC3H12D protein. This receptor protein is expressed on the surface of NK and T (CTL) cells, and their anti-tumor activities, including Granzyme B dependent tumoricidal activity, are enhanced by the synthetic short mRNA binding. Thus, synthetic short mRNA can be applied to treat lung metastasis. Part of the image is taken from TogoTV (^©^ 2016 DBCLS TogoTV, CC-BY-4.0 https://creativecommons.org/licenses/by/4.0/deed.ja.

There is only one case that has been reported so far. A 50-mer RNA, which is part of the 3’UTR in IL1β-mRNA, was first discovered to be an anti-metastatic short mRNA (19). In the study of anti-metastatic short mRNA, it was found that IL1β-mRNA is released from the host cells when stimulated by molecular signals, including inflammatory cytokines, derived from the tumor cells. Detailed analysis using *in vitro* and *in vivo* assays determined the functional core (50-mer) of IL1β-mRNA. The molecular signal of the 50-mer is sensed by the RNA-binding protein, ZC3H12D, on the surface of NK-type immune cells (19). These cells were found to be B220^+^CD11c^+^NK1.1^+^ in mice and a part of the CD3^-^CD56^+^ cell population in humans. The mouse B220^+^CD11c^+^NK1.1^+^ cells originally reside in the liver but relocate to the lungs (86) upon receiving signals from the primary tumor, undergoing changes in gene expression profile (87). Additionally, the fact that biological functions of extracellular IL1β-mRNA are ZC3H12D-dependent is confirmed by ZC3H12D knockout mouse study. In the lungs, the cells are activated by the extracellular mRNA to remove pathogenic extracellular protein aggregates containing citrullinated fibrinogen complexes, that accumulate in the pre-metastatic niche (88, 89). The pre-metastatic niche, which is interpreted as a highly probable metastatic site because it facilitates tumor metastasis before circulating tumor cells reach the metastatic location. It is formed by the tumor cell-derived molecular signals. Therefore, removing the pathogenic protein aggregations leads to the dissociation of the pre-metastatic niche. This indicates that short mRNA-primed NK subpopulations can prevent tumor metastasis (88, 90) (**Figure 3**).

## 6 Comparison of PK/PD profile between RNA drugs

As mentioned above, RNA drugs are rapidly eliminated from the body via renal filtration and liver accumulation. Therefore, extrahepatic tissue delivery requires a high dosage. Although RNA drugs are not metabolized by CYP450 enzymes, they are digested by nucleases. However, chemical modifications such as 2’-OMe can protect them from enzymes, and most RNA drugs are RNase resistant because of this. The concentration of RNA drugs in the blood decreases rapidly, but once inside the cell, they are expected to have a prolonged effect. Several studies demonstrated the administration of RNA drugs in mouse. Tail vein injection of siRNA (1–2 kg/mg) resulted in 50-80% knockdown of the target gene (Usp9x) in the livers of male mice 5 days after injection, with no significant increase in ALT levels (91). Subcutaneous or intratracheal injection of ASO (25 mg/kg subcutaneously or 1 mg/kg for intratracheally) resulted in a 50% reduction in the expression of the target gene (Malat1) at 4 weeks (92). Thus, a biological effect is expected when ~1 mg/kg of an RNA drug is administered to a mouse model. This assumption is consistent with data on IL-1β-mRNA 50-mer. The chemically modified IL-1β-mRNA 50-mer with 4’-aminoethyl and 2’-F at both ends and 2’-OMe in between, was detectable 30 min after injection, but not after 48 hours. A small amount of fluorescently labeled IL-1β-mRNA 50-mer was detected in tissue retrieved 24 hours after injection (25). Three doses of intravenous IL-1β-mRNA 50-mer (0.04 mg/kg per injection) blocked lung metastasis with no apparent severe side effects, as indicated by blood ALT, AST, and inflammatory cytokine levels. These results suggest the prolonged effects and low immunogenicity of IL-1β-mRNA 50-mer (25).

## 7 Conclusion

The field of synthetic short RNA is growing. Regarding ~100 mer RNA, the synthesis methodology is well-established and relatively simple, and its sequence variations can create a varsatility of target molecules. The sequence of synthetic short RNA can be designed to maximize hybridization-dependent or -independent functions, or a combination of the two, which cannot be generated by other modalities, such as antibodies. Due to these benefits, more therapeutic synthetic short RNAs will be developed in the future. The largest obstacle to RNA-based therapy is delivering it to tissues other than the liver. This issue can be resolved by improving the adduct molecule to recognize tissue-specific markers. The next generation of synthetic short RNAs will be aptamers and synthetic short mRNA. For these drugs, protein-RNA interactions are key in determining their effectiveness. Thus, the importance of RNA structure is increasing, as is the reliability of computational methods, which has grown rapidly since the advent of deep learning algorithms. Although the study of synthetic short mRNA is in its early stages and IL1β-mRNA is currently the only example, more synthetic short mRNA is expected to be developed in the future because extracellular mRNA is highly diverse, and each tissue may have its own mRNA signaling system including receptor proteins. Since cancer treatment remains challenging, we anticipate that new anticancer modalities, aptamers and synthetic short mRNA, will be explored.
